# Variations of deep soil moisture under different vegetation restoration types in a watershed of the Loess Plateau, China

**DOI:** 10.1038/s41598-023-32038-0

**Published:** 2023-03-27

**Authors:** Tingting Meng, Pei Sun

**Affiliations:** 1 Shaanxi Provincial Land Engineering Construction Group Co., Ltd., Xi’an, China; 2 Institute of Land Engineering and Technology, Shaanxi Provincial Land Engineering Construction Group Co., Ltd., Xi’an, China; 3 Key Laboratory of Degraded and Unused Land Consolidation Engineering, Ministry of Natural Resources, Xi’an, China

**Keywords:** Solid Earth sciences, Sedimentology

## Abstract

The soil water content and water consumption of deep layer (200–1000 cm) of sloping farmland, grassland and Jujube orchard in Yuanzegou small watershed in the loess hilly region were studied. The results showed that (1) the soil moisture content of sloping farmland, grassland and Jujube orchard increased at first and then decreased at 0–200 cm, with mean values of 11.91%, 11.23% and 9.99% respectively; From 200 to 1000 cm, the soil moisture content decreased slowly and tended to be stable, with mean values of 11.77%, 11.62% and 9.96% respectively. (2) 200–1000 cm, the soil water storage ranged in the order of sloping farmland > grassland > Jujube orchard, with mean values of 148.78, 145.28 and 121.11 mm respectively. (3) In the 200–1000 cm soil layer, the water consumption of the Jujube orchard ranged from 21.67 to 32.97 mm, and that of grassland ranged from − 4.47 to 10.32 mm, the water consumption of deep soil in Jujube orchard was significantly higher than that in grassland (*p* < 0.05)., Although the Jujube orchard had obvious deep soil moisture consumption, it was insufficient to cause serious soil drying and increased farmers' income, so it can be planted locally, but the planting density should be reasonable and water-saving engineering technology should be adopted.

## Introduction

Change to the land surface is an important driver of eco–hydrological change^1–5^. The effects of changes to land use/land cover (LULC) on hydrological processes and water resources in the basin are mainly reflected in surface runoff, runoff, evapotranspiration (ET) and soil moisture^6–8^. Especially, soil moisture in different soil layers is usually related to different hydrological processes and ecological functions^9^. Surface or shallow layer soil moisture is usually greatly influenced by rainfall infiltration or evapotranspiration and is a regular water source for vegetation growth, while the moisture in deep soil layers functions as a soil reservoir^10^. In arid and semi-arid regions, soil moisture has become an important limiting factor for vegetation growth. Deep soil moisture, as a reserve water resource for plant growth and utilization, plays a vital role in the response of plants to extreme weather events such as long-term drought^11,12^. Such as the Loess Plateau of China, where water resources are incredibly scarce, in such regions, deep soil moisture even becomes the main constraining factor of plant productivity and ecosystem sustainability^13,14^.

The Loess Plateau of China is located in a semi-arid region with low annual rainfall (150–600 mm) and high evaporation (1400–2000 mm)^15^, resulting in drought and low soil water content in this region. A range of comprehensive afforestation programs have been initiated by the Chinese government to improve ecosystem services and conserve soil in the region. These include the “Grain-for-Green Program (GFGP)” in the 1990s^16^, dramatically changing the landscape. Introduced vegetation reduced soil erosion and increases carbon storage. But, it has been reported that large-scale vegetation restoration caused runoff attenuation; the increase in water consumption due to vegetation transpiration will reduce the recharge of precipitation to groundwater, and the soil moisture^17–19^. This will lead to an increase in regional aridity and aggravate the burden of water resource utilization. In addition, due to the thick loess soils exceeding 30 m in depth and low annual precipitation (< 600 mm), the shallow soil moisture is not sufficient to meet the growth needs of introduced vegetation^20^. To absorb deeper soil moisture, the forest species and perennial grass that have established themselves over the last 30 years extending their roots to a depth exceeding 10 m^21^, leading to a large amount of deep soil moisture consumption, even causes soil dry layer.

At present, studies focusing on the impacts of restoration of vegetation on soil moisture in loess hilly-gully regions are mainly carried out from the perspectives of to-pography, land use, planting density, introduced species. For example, Yu et al.^22^ studied the soil moisture under a 0–5 m soil depth in the semi-arid LP, and found that afforestation caused a decrease in soil moisture of deep soil, especially on the hillside, and the content of forest soil moisture was lower than that of grassland and farmland. Yang’s study^23^ in the Longtan Watershed of Dingxi City, Gansu Province, showed that slope position and aspect mainly affected shallow soil moisture, while gradient and slope mainly affected deep soil moisture. Yang et al. showed that after the conversion of traditional farmland on the semi-arid was converted into grassland, shrub, and forest land, soil moisture at a depth of 2 m decreased by more than 35%, a soil water deficit occurred in areas of introduced vegetation^24^. Research^25^ shows that shrub and grass consume more than abandoned land and farmland in terms of soil moisture, and suggests that native grass is the optimal crop-land cover species in the region. Other studies^20,26,27^ found that introducing vegetation into the LP was the main factor causing soil moisture reduction. The soil depth determined by the above research is primarily concentrated in the shallow soil layer, unable to clearly reveal the sustainable needs of vegetation restoration. In addition, these studies emphasize that the main cause of soil water deficit is introduced vegetation type, and there is less research on soil water change of due to native cash- crop types that can increase farmers' income.

After the implementation of the project of "GFGP" in the LP, the planting area of Jujube tree, apple and other economic tree species, as traditional drought-tolerant cash crops, increased sharply in the loess hilly region, which contributed to local ecological restoration and increased the income of local residents. The Jujube (Ziziphus Jujuba Mill) is a fruiting tree species with drought resistance and great economic potential that has been cultivated for approximately 2000 years on the LP of China. Over the past decade, The area of Jujube plantations on the LP has been reached 122·376 ha due to both economic and ecological policy goals^28^. Unlike ecologically planted species, economically planted forests are generally strictly managed by the landowner (via fertilization, pruning, etc.). Therefore, there is a need for further analysis of the dynamic change in soil moisture in artificial economic forests, so as to understand the effect of human management activities on soil moisture.

Therefore, we aim to understand the relationship between the native vegetation types and the dynamic changes of deep soil moisture, and assume that the jujube forest in the study area will not lead to soil drying as a 10-year economic forest. We studied the effect of vegetation on the dynamic changes in water content in deep soil (200–1000 cm) in the LP after returning farmland to forest and grassland. The specific research objectives were as follows: (1) identify vertical distribution of soil particle composition and organic carbon; (2) identify vertical distribution of soil moisture content (3) identify vertical distribution of water consumption in deep soil; (4) identify factors affecting deep soil moisture.

## Materials and methods

### Overview of the study area

The study area of the present study was the Yuanzegou watershed in Shaanxi Province (37° 15’ N, 110° 21’ E). This basin is composed by complex features: gullied slopes of hills (20° < mostly with gradients < 45°) in upper parts and deep, the loess soils are typical silt loams belonging to Inceptisols (United States Department of Agriculture), usually with > 50% silt contents and < 30% clay contents. This watershed is a typical loess hilly gully basin extending over an area of 0.58 km^2^, with the gully watershed covering 0.31 km^2^ (53.4% of the area of the total watershed). The climate in the study area is temperate continental monsoon. Average annual rainfall is 505 mm, with most of it occurring within 7–9 months, an average annual temperature of 8.6 °C, with the lowest and maximum monthly air temperatures of − 6.5 °C and 22.8 °C, occurring in January and July respectively.

A large area of sloping farmland in the watershed has been transformed into natural restored grassland and artificial Jujube orchard since returning agricultural land to forest and grassland. As a traditional drought-tolerant economic species, the planting area of artificial Jujube orchards has increased sharply in the region, making a significant contribution to local ecological restoration and increasing the economic income of local residents. In the watershed, sloping farmland, artificial Jujube orchard and natural restoration grassland are the most common land types. The sloping farmland mainly grows millet (*Setaria italica*) and maize (*Zea Mays*). The main variety of Jujube in the orchards is Junzao (*Ziziphus Jujuba Mill*), the management is mainly via clear tillage, with no artificial fertilization or irrigation. The main vegetation types on the natural restored grassland are shallow root plants with small size and canopy, such as *Tripolium vulgare*, *Stipa bungeana, Trin and Artemisia scoparia*, which grow on the ground without human interference. The biological characteristics of Jujube orchard plants are shown in Table 1.

**Table 1:** The biological characteristics of Jujube orchard plants.

Trees species	Planting method	Average height (m)	Average diameter at breast height (mm)	Average diameter crown (m)
Ziziphus jujuba Mill	Monoculture species	2.47 ± 014	75.36 ± 9.57	1.86 ± 0.21

### Research methods

In May 2020, 10 years of reclaimed grassland and 10 years of reclaimed Jujube orchard were chosen for analysis, with data for sloping farmland taken as the control. The slope direction and slope were approximately as same, and soil samples were randomly taken using a soil auger with a diameter of 40 mm. The depth of the soil was 1000 cm. The 0–200 cm soil layer was considered shallow soil and was separated into 20 cm layers. Starting from 200 cm, soil was separated into 100 cm layers. Portable handheld GPS (MG838, Uni Strong) was used to record the detailed geographic information of the samples, and basic information is shown in Table 2. Three sub-subsamples were taken from random positions around each sample point, and after evenly mixing in each layer, sundries were picked out, some of which were put into plastic sealed bag and some into aluminum boxes. The soils in aluminum boxes were oven dried for 24 h at 105°. A weighing method was used to measure soil moisture^29^. The soils in the plastic sealed bags were dried by natural air, and screened with 2 mm and 1 mm sieves respectively. The soil samples sieved by 2 mm were used to measure soil mechanical composition. Soil texture classification was based on the American agricultural system to classify soil particle size: clay (< 0.002 mm), silt (0.002–0.05 mm) and sand (0.05–2 mm), which were determined by a Malvern Laser Particle size analyzer (Mastersizer2000, Malvern Instruments Ltd). The mass fraction of soil organic carbon was determined using a 1 mm sieved soil and the potassium dichromate volumetric technique^30^.

**Table 2:** Basic information of the study area.

Sample plot	Elevation/m	Slope/°	Aspect/°	Vegetation types
Sloping farmland	954.3–1015.0	3.2–31.6	11.6–344.6	*Setaria italic, Zea mays*
Grassland	930.8–1019.2	21.7–46.7	15.8–335.0	*Tripolium vulgare*
Jujube orchard	962.4–1066.3	18.5–47.7	2.5–341.8	*Ziziphus jujuba Mill*

### Data analyses

The soil moisture content in the 0–200 cm layer is heavily influenced by precipitation infiltration and vegetation evapotranspiration^23^. So, the present study defined the soil layer at a depth exceeding 200 cm as deep soil, and the effect of returning agricultural land to forest or grassland on deep soil moisture was discussed in the depth range of 200–1000 cm. The calculation formula of soil water storage is:

1 $${\text{S}}_{ms} = {\text{w}}*\rho *{\text{H}}/{1}0,$$

where S_*ms*_ is soil water storage (mm); *w* is soil moisture (%); *ρ* is the bulk density of soil (g/cm^3^); *H* is the depth of soil (cm).

The bulk density (*ρ*) of the 200–1000 cm soil layer was approximated using the phase transfer function (PTF) established by Wang Yunqing^31^ based on the LP data set.

2 $$\begin{aligned} \rho_{i} = & {1}.{8284} + 0.0{429}*{\text{log}}_{{{1}0}} {\text{Clay}}_{{\text{i}}} + 0.0{2}0{5}*{\text{Clay}}_{{\text{i}}}^{{0.{5}}} \\ & - 0.0{125}*{\text{cosClay}}_{{\text{i}}} - 0.00{61}*{\text{Silt}}_{{\text{i}}} + 0.000{1}*{\text{Silt}}_{{\text{i}}} *{\text{SGi}} \\ & - 0.00{9 8}*{\text{SG}}_{{\text{i}}} - 0.00{71}*{\text{SOCi}} - 0.0{5}0{5}*{\text{SOC}}_{{\text{i}}}^{{0{5}}} + 0.000{2}*{\text{SOC}}_{{\text{i}}}^{{0.{5}}} , \\ \end{aligned}$$

where Clay_*i*_ and Silt_*i*_ refer to the volume fraction of clay particles and silt particles, respectively , at layer *i*, SG_*i*_ refers to the slope of each sample point, and SOC_*i*_ refers to the mass fraction of soil organic carbon at layer *i*.

The soil water consumption is the initial soil water storage minus the existing water storage, and the water storage of control land (sloping farmland) is taken as the initial water storage. The calculation formula is:

3 $${\text{W}}_{{{\text{depete}}}} = {\text{ S}}_{{{\text{initial}}}} - {\text{ S}}_{{{\text{present}}}} ,$$

where *W*_depete_ is soil water consumption (mm); *S*_initial_ is the water storage of slope farmland (mm); *S*_present_ is the amount of water currently stored in the soil (mm).

Excel2020 and Spss22.0 software were used for data analysis, and Origin2018 software was used for mapping. One-way ANOVA was used to study the different factors affecting on deep soil moisture content, and a comparison was made against the LSD method. The present study used Pearson correlation analysis to quantify the relationship between deep soil moisture and texture of soil.

## Results and analysis

### Composition of soil particles and organic carbon distribution

**Figure 1:**
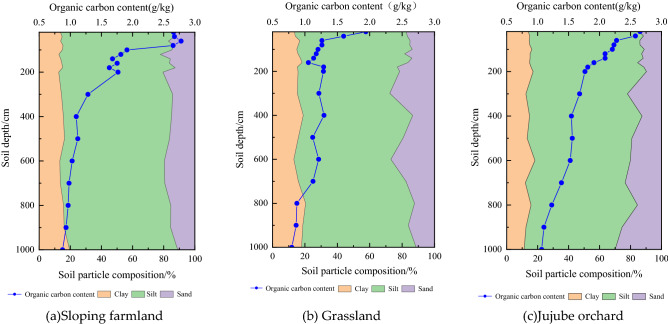
Vertical distribution of soil particle composition and organic carbon mass fraction. Sloping farmland (**a**), Grassland (**b**), Jujube orchard (**c**).

Soil particle composition and organic carbon distribution profiles at 0–1000 cm Soil layer for sloping farmland, grassland and Jujube orchard are shown in Fig. 1. The variation range of the soil clay volume fraction was 12.55–19.51%, 13.17–20.4% and 11.27–18.24% in sloping farmland, grassland and Jujube orchard, respectively. The average silt volume fraction of particles in these regions ranged from 65.19% to 72.33%, 57.08% to 71.31% and 58.8% to 73.41%, respectively. The average sand volume fraction varied from 11.05% to 22.20%, 11.38% to 27.63% and 11.13% to 29.92%, respectively. The magnitude of change in clay volume fraction was in the order of Jujube orchard < sloping farmland < grassland, the change trend of silt volume fraction was grassland < jujube orchard < sloping farmland, and that of sand volume fraction was in the order sloping farmland < Jujube orchard < grassland.

The soil organic carbon content of the sloping farmland, grassland and Jujube orchard decreased with increasing soil depth. In the shallow 0–200 cm soil layer, soil organic carbon (SOC) content in sloping farmland, grassland and Jujube orchard was 2.13, 1.32, and 2.16 g/kg, respectively. In the 200–1000 cm soil layer, soil organic carbon content decreased slowly and tended to be stable, with mean values of 1.12, 1.09, and 1.43 g/kg, respectively. SOC content in the 0–1000 cm layer was in the order of Jujube orchard > sloping farmland > grassland.

### Depth variation in soil moisture content

**Figure 2:**
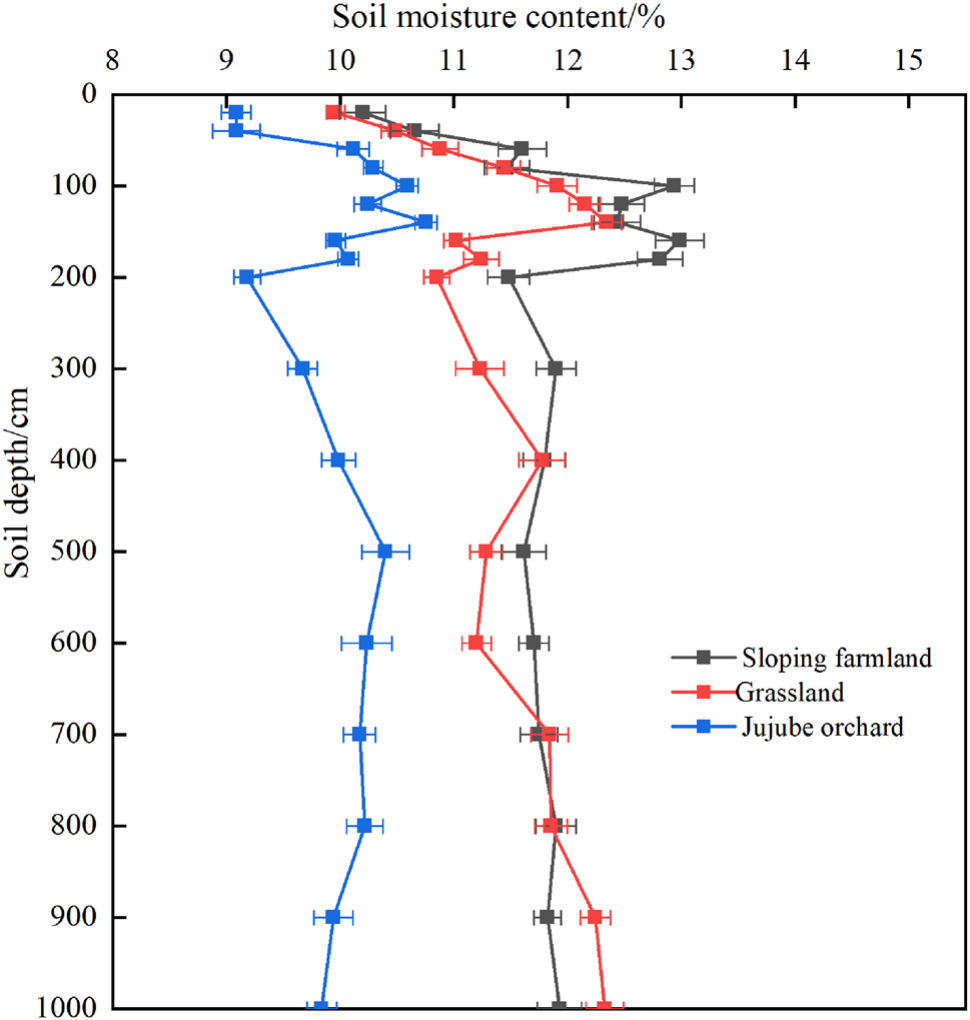
Soil depth distribution of soil moisture.

Figure 2 illustrates the depth variation in soil moisture content in sloping farmland, grassland, and a Jujube orchard. The soil moisture content in the 0–200 cm soil layer showed a trend of increasing first and then decreasing, and fluctuated greatly. The range of soil moisture content in sloping farmland was 10.20–12.99%, with an average of 11.91%. The range of soil moisture content in grassland was 9.96–12.15%, with an average of 11.23%. The soil moisture content of Jujube orchard varied from 9.10 to 10.75%, with an average of 9.99%.

At a depth of 200–1000 cm, the soil moisture content of sloping farmland decreased slowly and tended to be stable, and the overall fluctuation of soil moisture was small, ranging from 11.40 to 11.93%, with an average of 11.77%. The grassland soil moisture content increased in the 900–1000 cm soil layer, with a range of 10.85% to 12.33% and an average of 11.62%. The soil moisture content of Jujube orchard increased slightly in the 300–500 cm layer, but decreased steadily in the 600 cm layer, with a range of 9.18–10.40% and an average of 9.96%. There were significant differences in soil moisture contents of jujube orchard and grassland with increasing soil depth within a range of 300–1000 cm.

### Soil depth distribution characteristics of water consumption

**Figure 3:**
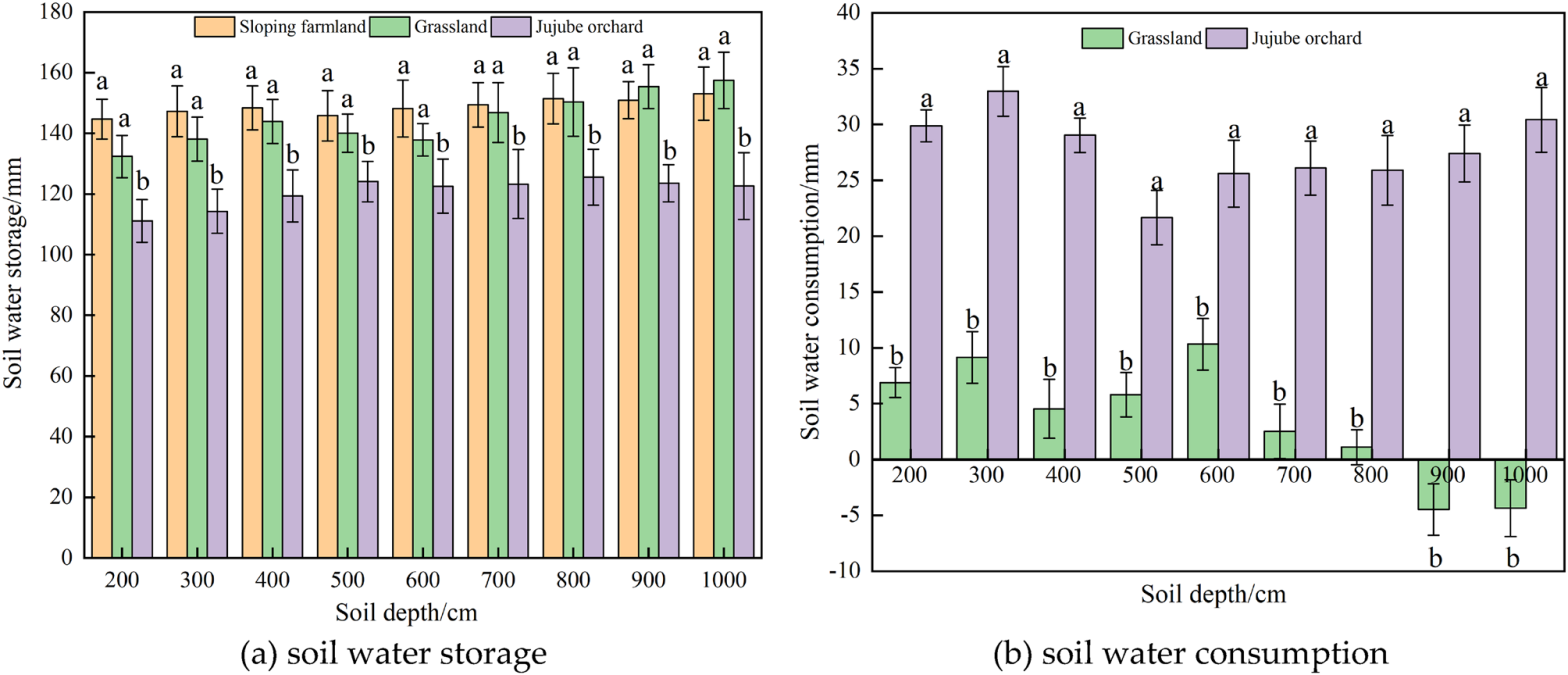
Vertical distribution of the deep soil water storage (**a**) and soil water consumption (**b**).

The soil water storage of sloping farmland, grassland and Jujube orchard is illustrated in Fig. 3a. At a soil depth of 200–1000 cm layer, the soil water storage in the sloping farmland varied from 144.67 to 153.06 mm, with an average of 148.78 mm; with increasing of soil depth, the soil water storage showed little difference. The range of grassland water storage was 137.78–157.42 mm, with an average of 145.28 mm; water storage was positively correlated with soil depth. The water storage of Jujube orchard ranged from 114.29 to 125.53 mm, with an average of 121.11 mm; there was little difference with the increase in soil depth. Soil water storage in sloping farmland and grassland was significantly higher than that in Jujube orchard (*P* < 0.05), but there was no significant difference in soil water storage between sloping farmland and grassland. The average soil water storage in the 200–1000 cm layer was in the order of was sloping farmland > grassland > Jujube orchard, but in the 900 cm and 1000 cm soil layer, the soil water storage in grassland was slightly higher than that in sloping farmland.

Figure 3b shows the consumption of deep soil moisture in the grassland and Jujube orchard. At a depth of 200–1000 cm, the consumption of deep soil moisture in the Jujube orchard significantly exceeded that of grassland (*P* < 0.05). The deep soil moisture consumption of the Jujube orchard ranged from 21.67 to 32.97 mm, with an average of 27.67 mm. The lowest water consumption occurred in the 500 cm soil layer (21.67 mm), the highest water consumption occurred in the 300 cm soil layer (32.97 mm), and no significant difference in the water consumption in the 600–900 cm soil layer was detected. With increasing soil depth, the water consumption of the Jujube orchard showed no obvious change. The deep soil moisture consumption of the grassland ranged from − 4.47 to 10.32 mm, with an average of 3.50 mm. The water consumption of the grassland varied greatly among different soil layers, and the water consumption at the 300 cm and 600 cm soil layers was slightly higher than that of other soil layers (9.14 mm and 10.32 mm, respectively). From the soil layers of 700 cm to 900 cm and 1000 cm, the deep soil moisture consumption of the grassland showed a decreasing trend, until 900 cm and 1000 cm when the soil water consumption showed a negative value of − 4.47 mm and − 4.35 mm, respectively, that is, there was no water consumption. The deep soil moisture consumption of grassland decreased with increasing soil depth.

### Influencing factors of deep soil moisture content

Table 3 shows the correlation between soil moisture content and particle composition and organic carbon. There was a significant positive correlation between deep soil moisture and clay content of sloping farmland, grassland and Jujube orchard. There was a significant negative correlation between deep soil moisture and sand content in the sloping farmland, and a significant negative correlation between deep soil moisture and sand content in the grassland and Jujube orchard. There was a negative correlation between deep soil moisture and silt content of the sloping farmland and Jujube orchard, and a positive correlation between soil moisture content and silt content in grassland. There was a negative correlation between the deep soil moisture and organic carbon content in the sloping farmland and grassland, and a significant positive correlation between the deep soil moisture and organic carbon content in the Jujube orchard.

**Table 3:** Correlation between soil moisture content and particle composition and organic carbon in deep soil of study area.

Sample plot	Clay	Silt	Sand	Organic carbon
Slope farmland	0.50**	− 0.33	− 0.86**	− 0.18
Grassland	0.43**	0.32	− 0.55*	− 0.15
Jujube orchard	0.40**	− 0.21	− 0.49*	0.33*

"*" in the table represents the significance level at 0.05, and "**" represents the significance level at 0.01.

## Discussion

Due to the deep loess layer of the Loess Plateau, the depth of rainfall infiltration is not more than 2 m^9,19^, which makes the water content of the shallow 0–200 cm soil greatly affected by rainfall and evapotranspiration. In addition, the ground vegetation also has a great impact on rainfall interception and infiltration^32,33^. Therefore, the soil moisture of sloping farmland, grassland and Jujube orchard in this study fluctuates greatly in 0–200 cm soil layer, showing a trend of increasing first and then decreasing, which is consistent with previous studies on the Loess Plateau^34,35^.In this study, soil moisture content and water storage are highest in the sloping farmland. These observations are consistent with those previously reported for the soil moisture changes under different vegetation types in the Loess Plateau^22,24,36^, which have been shown that the content of soil moisture of agricultural land significantly exceeded those of the other vegetation types. In addition, it also showed that soil planted on gentle slopes in a small watershed of a loess hilly-gully region has better water storage capacity, which e similar to Xin's findings^37^. The main reason for this is that the slope of sloping land is above 15°, and there is no mechanical ploughing. Thus, the disturbance of land is small and soil erosion is reduced. Factors involved include long-term cultivation, soil surface loss, large porosity, conductivity to rainfall infiltration, the main crops being corn and millet, the root system being shallow, ripe years, and less water consumption. The application of organic fertilizer to divert water has a certain effect on soil moisture retention^38^.

In this study, the soil moisture content and water storage of grassland are higher and only second to that of sloping farmland, with little difference from that of sloping farmland. This was mainly because the grassland in this study was a native herb, and the water consumption was low. This result is in accordance with the results of Xiao et al.^39^ in Zhifanggou of Ansai in a loess hilly region, which showed no significant difference in the contents of water between natural grassland (Artemisia argyi) and sloped farmland (Setaria italica). This is also similar to Fang's study in the Ansai Watershed of Loess High Plateau^10^, which showed that soil moisture content in the 0–500 cm soil layer is higher in farmland (water content 11.07–11.79%) and natural grassland (10.5–11.19%). However, in contrast to these results from artificial grassland, previous studies^40,41^ showed that the soil moisture content of artificial grassland was lower as a result of a deep distribution of roots and large biomass (such as LotuscorniculatusL). In this study, although deep soil water consumption occurred in the grassland, the water consumption was relatively low, ranging from − 4.47 to 10.32 mm not enough to cause a serious soil water deficit in the dry layer and the region. The main reason for this is that the grassland vegetation in the study area involves naturally recovered plants with shallow root systems, such as Artemisia gmelinii, with a small volume and canopy. The vertical root system is not developed, but the horizontal root system is^42^. On the one hand, the litter layer on the surface has a great capacity to hold water, which can effectively increase the amount of surface water infiltration. On the other hand, developed horizontal roots can also effectively improve soil anti-scour and reduce soil erosion^43^.

In this study, the water consumption characteristics of Jujube orchards are the same as those of other plantations, but the water consumption is very different. Yang et al.^44^ showed in their study on a loess hilly-gully region that both artificial *Robinia pseudoacacia* forest and artificial Caragana korshinskii Kom displayed significant soil water consumption in the 200–500 cm soil layer, and soil desiccation occurred to varying degrees. However, there was no desiccation in barren grassland and cultivated land. Liu^45^ studied the variation deficit and recovery of deep soil water in apple orchards on the LP and found that the cumulative deficit of soil water demand in apple orchards at a depth of 3–18 m could reach 1200 mm, resulting in a severe water deficit in apple orchards. Li^46^ found in his research on the LP of northwest Shanxi Province that when rainfall was less than 405 mm, the deep soil of *Robinia pseudoacacia* L. forest would be short of water, forming a dry soil layer. Compared with the above studies, the water consumption of the Jujube orchard in the study area is 21.67–32.97 mm, which is relatively low and will not cause a dry soil layer. The main reason is that the artificial management of jujube orchard, such as reasonable planting density, application of organic fertilizer and long-term no-tillage system, has reduced soil water loss. This is similar to Fang's research^10^, due to the artificial rainwater collection measures, the soil in apple orchards did not dry out at 0–500 cm. Inappropriate planting of artificial vegetation on the LP was the cause of deep soil water loss, and appropriate vegetation types should be selected based on rainfall and soil moisture conditions^17^. Ten years after planting, Jujube orchard in the economic forest in this region did not cause a serious soil dry layer, and they increased farmers' income. This can be promoted locally, but a reasonable density value should be used in planting and water-saving engineering technology should be adopted, such as fish-scale pit technology, adopted by Li et al.^47^ in Jujube orchards on the LP.

The results of the present study showed clay content and sand content were significantly positively and significantly negatively correlated with the deep soil moisture of sloping farmland, grassland, and Jujube orchard, respectively, consistent with previous studies^10^, which shown that the texture of soil is the key factor affecting the deep soil water status. After returning farmland to forest and grass, the average clay and sand volume fractions of grassland increased, mainly because the litter on grassland surface formed humus in soil, which has a good protective effect on soil and increases clay volume fractions. At the same time, litter promoted soil ripening, improved soil structure, and increased the large particles (0.1–0.2 mm) in the soil, thus the volume fraction of grassland sand also showed an increasing trend.

Although we have demonstrated that there is significant deep soil moisture consumption in Jujube orchard and grassland in the study area, our understanding of soil moisture after returning farmland to forests and grasslands still has limitations. Multiple studies have shown that root characteristics also affect soil moisture content and water uptake in the deeper layers of secondary grasslands and forests^21,48^. Therefore, we suggest that root characteristics should be considered in future studies of soil deep moisture consumption of vegetation restoration.

## Conclusions

1. In this small watershed on the Loess Plateau, the average water content and water storage of the 0–1000 cm soil layer were in the range of sloping farmland > grassland > Jujube orchard. There was no significant difference between sloping farmland and grassland, but the values in the Jujube orchard was were significantly. In the shallow 0–200 cm soil layer, the soil moisture content and water storage fluctuated greatly, but tended to be stable in the 200–1000 cm soil layer.
2. In the 200–1000 cm soil layer, the Jujube orchard had displayed obvious deep soil moisture consumption, which was significantly higher than that of grassland. The deep soil moisture consumption of grassland was very low, and there was no water consumption in the soil layer of 900–1000 cm
3. Soil texture was one of the main factors affecting deep soil moisture. The deep soil moisture of sloping farmland, grassland and the Jujube orchard was significantly positively correlated with clay particles, but negatively correlated with sand particles. There was a negative correlation between soil deep moisture and organic carbon content in sloping farmland and grassland, and a significant positive correlation between deep soil moisture and organic carbon content in the Jujube orchard.
4. After 10 years of planting of jujube orchard, although there is deep soil water consumption, it does not cause dry soil layer. Therefore, our assumption is true that the planting of jujube orchard in this area will not cause dry soil layer. As a way to increase local economic income, jujube orchard is suitable for planting in this area, but planting should be carried out at a reasonable density, and reasonable water-saving engineering technology should be adopted.

## Data Availability

The datasets used and/or analysed during the current study available from the corresponding author on reasonable request.
